# Delivery of Probiotics with Cellulose-Based Films and Their Food Applications

**DOI:** 10.3390/polym16060794

**Published:** 2024-03-13

**Authors:** Ying Yang, Junze Zhang, Chengcheng Li

**Affiliations:** International Innovation Center for Forest Chemicals and Materials, Jiangsu Co-Innovation Center for Efficient Processing and Utilization of Forest Resources, Nanjing Forestry University, Nanjing 210037, China; realreallyyy@163.com (Y.Y.); 18959778670@163.com (J.Z.)

**Keywords:** cellulose, probiotic, encapsulation, food application

## Abstract

Probiotics have attracted great interest from many researchers due to their beneficial effects. Encapsulation of probiotics into biopolymer matrices has led to the development of active food packaging materials as an alternative to traditional ones for controlling food-borne microorganisms, extending food shelf life, improving food safety, and achieving health-promoting effects. The challenges of low survival rates during processing, storage, and delivery to the gut and low intestinal colonization, storage stability, and controllability have greatly limited the use of probiotics in practical food-preservation applications. The encapsulation of probiotics with a protective matrix can increase their resistance to a harsh environment and improve their survival rates, making probiotics appropriate in the food packaging field. Cellulose has attracted extensive attention in food packaging due to its excellent biocompatibility, biodegradability, environmental friendliness, renewability, and excellent mechanical strength. In this review, we provide a brief overview of the main types of cellulose used for probiotic encapsulation, as well as the current advances in different probiotic encapsulating strategies with cellulose, grafted cellulose, and cellulose-derived materials, including electrospinning, cross-linking, in-situ growth, casting strategies, and their combinations. The effect of cellulose encapsulation on the survival rate of probiotics and the patented encapsulated probiotics are also introduced. In addition, applications of cellulose-encapsulated probiotics in the food industry are also briefly discussed. Finally, the future trends toward developing encapsulated probiotics with improved health benefits and advanced features with cellulose-based materials are discussed.

## 1. Introduction

Probiotics are live microorganisms that exert beneficial effects on their host, when administered in adequate amounts [1]. They are widely used to produce dairy products, such as yoghurt and drinks, as well as functional foods. Currently, various forms of probiotic dietary supplements have been developed, including probiotic powders, probiotic candies, probiotic capsules, etc. [2]. With the increase in health awareness and the flourishing of probiotic-related research, various probiotic-related industries are developing rapidly, and the sales of the probiotic products are expected to grow at an annual rate of approximately 7.4% [3]. Probiotics exert beneficial effects by producing functional metabolites, modulating intestinal microecology, regulating the immune system, and preventing pathogens [4]. However, it is difficult for probiotics to reach the intestinal tract in adequate amounts and activity due to the susceptibility of probiotics to adverse conditions such as temperature, oxygen, water activity, pressure, pH, hydrogen peroxide, and digestive enzymes during processing, storage, and digestion, thus affecting the beneficial effects of probiotics [5]. In addition, during processing and storage, probiotics are subjected to high temperatures, high acidic or alkaline conditions, and other harsh conditions, which result in a significant reduction in the viability of probiotics [6]. Therefore, it is necessary to maintain the high viability of probiotics during processing, storage, and digestion to promote their efficacy. Probiotic encapsulation is an effective strategy for protection and efficient delivery of probiotics during processing, storage, and oral administration.

Natural polymer-based materials (e.g., polysaccharides, proteins, and lipids) are biocompatible, food-grade, biodegradable, and widely available, making them ideal matrixes for protection and controlled delivery of probiotics [7]. For example, proteins, including soybean protein, whey protein, and soybean protein isolate (SPI), etc., are used as encapsulation materials for probiotics due to their low cost, abundant sources, and high nutritional properties [8]. However, proteins typically encapsulate probiotics by interacting with a variety of active compounds or other polymers via their surface functional groups [9,10,11,12]. Lipids can also be used to encapsulate probiotics [13,14]. Lipids are often used to encapsulate probiotics by forming different types of emulsions or forming lipid membranes by self-assembly under the action of calcium ions [15,16]. Compared with proteins, encapsulation of probiotics using lipids is less studied, except for emulsions. Polysaccharides (e.g., cellulose, alginate, chitosan, and gellan gum) are commonly used as probiotic encapsulation materials due to their biocompatibility, low-cost, accessibility, modifiability, and pH-responsive properties. They can be used alone or combined with other materials for probiotic encapsulation to improve the survival and colonization rates of probiotics in the intestine, to realize the targeted release in the intestine, and to guarantee the retention time of probiotics in the gut. Among the polysaccharides, cellulose is one of the most used protective matrices for encapsulating probiotics to improve the survival rate of probiotics in harsh environments, to maintain the viability of probiotics, and to extend the storage time of probiotic products [17,18]. Various cellulose derivatives, such as hydroxypropyl cellulose and carboxymethyl cellulose (CMC) have been extensively used as probiotic encapsulation materials [19,20]. The cost of cellulose production is much lower than that of other materials, making it ideal for encapsulating probiotics by creating a microenvironment that protects probiotics from harsh environments and maintains the metabolic activity of probiotics. In addition, cellulose can maintain the viability of probiotics for a long time during storage. Moreover, cellulose encapsulation of probiotics has the advantages of safety, non-toxicity, high reactant resistance, high biomass density, high activity, and stability. When cellulose-encapsulated probiotics are used as a starter culture for yogurt fermentation, the stability of the released probiotics in the fermented milk is significantly improved, and the cellulose-based starter culture can be reused several times, enabling recyclability, long-term fermentation, and biotransformation [21].

**Figure 1:**
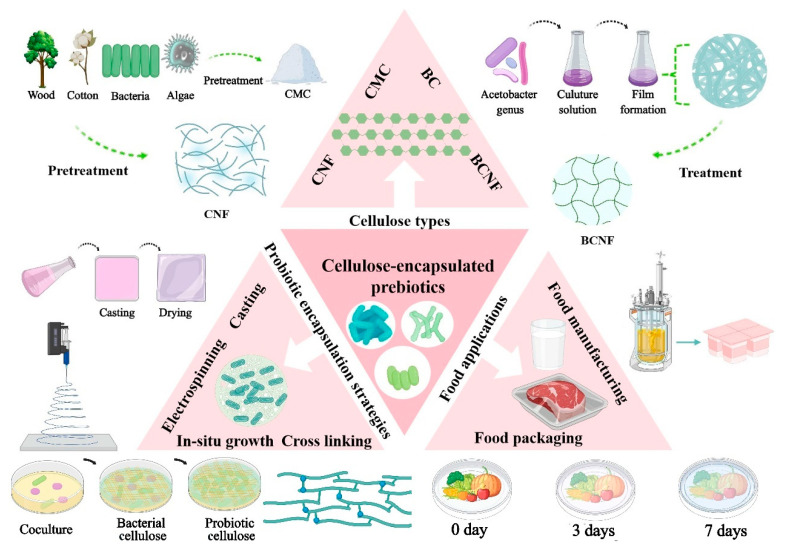
Schematic illustration of the types of cellulose used for probiotic encapsulation, probiotic encapsulating strategies with cellulose or cellulose-based materials, and the applications of encapsulated probiotics in the food field.

In this review, we will comprehensively summarize the advances in the development of cellulose-encapsulated probiotics for food applications from five aspects: (1) the types of cellulose used for encapsulating probiotics, (2) strategies for probiotic encapsulation using cellulose or grafted cellulose, (3) the effects of cellulose encapsulation on the survival rate of probiotics, (4) patented encapsulated probiotics, and (5) the applications of cellulose-encapsulated probiotics in food industry (Figure 1). Finally, the current challenges regarding the cellulose-encapsulating probiotics and future prospects in this field are proposed. We hope that this review can have implications for further research on designing and developing cellulose or cellulose-based materials for encapsulating probiotics and promoting their practical applications.

## 2. Cellulose Types Used for Probiotic Encapsulation

Cellulose is globally abundant as a major component of the plant cell wall. Recently, cellulose/cellulose-based materials have attracted extensive attention due to the biocompatibility, biodegradability, renewability, environmental friendliness, functionalizability, and excellent mechanical strength [22,23]. They have been extensively studied in various fields, including packaging, electronics, healthcare materials, printing, and materials science [24,25]. Many reviews about the types, preparations, modifications, and applications of cellulose have been published [26,27,28]. In addition, although there are many types of cellulose and cellulose derivatives that have been developed and applied in various fields, not all cellulose and cellulose derivatives are studied for the use in encapsulating probiotics. Thus, in this section, we will briefly introduce several representative celluloses for encapsulating probiotics, including bacterial cellulose (BC), bacterial cellulose nanofibers (BCNF), carboxymethyl cellulose (CMC), and cellulose nanofiber (CNF) from the aspects of their production methods, characteristics, and effects on the survival rates of probiotics before and after cellulose encapsulation (Table 1). Since cellulose is an indigestible fiber that is resistant to gastric juices, probiotics have an enhanced adverse environmental tolerance and survival rate after cellulose encapsulation. So, cellulose is particularly suitable as a material for protecting and controlling the delivery of probiotics.

### 2.1. Bacterial Cellulose (BC)

**Figure 2:**
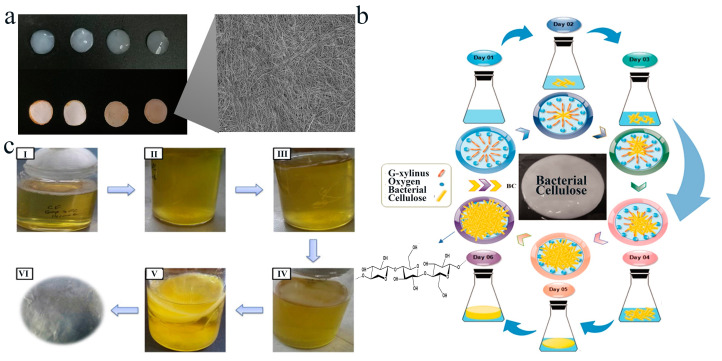
Morphology and production of BC. (**a**) Photographs of the freshly prepared BC film (**left top**), freeze-dried BC film (**left bottom**), and a scanning electron microscope (SEM) image of the BC film (**right**). (**b**) Schematic illustration of the bacterial cellulose preparation process by the bacterial cultivation method. Adapted from Ref. [57] with permission of SAGE Publications Ltd., 2022. (**c**) Illustration of the BC production process via the cell-free system. (I) Cultivation of cell-free enzyme solution with glucose source as carbon source, (II) formation of fibrils, (III) self-assembly of fibrils, (IV) formation of bio-cellulose pellicles, (V) formation of bio-cellulose pellicles, and (VI) harvesting of bio-cellulose pellicles. Adapted from Ref. [59] with permission of Elsevier Ltd., 2015.

BC is a polymeric material produced by aerobic bacteria, such as *Acetobacter xylinum* (*A. xylinum*), *Gluconacetobacter xylinus* (*G. xylinum*), and *Komagataeibacter xylinus* (*K. xylinum*), etc. [39]. The Food and Drug Administration (FDA) has approved BC as “generally recognized as safe (GRAS)” since 1992, and it is an insoluble dietary fiber [40]. BC represents the purest form of cellulose, without any pectin, lignin, hemicellulose, or arabinose [41]. It has attracted much attention in different fields such as biomedicine [42,43,44], textiles [45], food [46], 3D printing [47], and cosmetics [48] due to its unique properties, such as ultra-fine three-dimensional (3D) fibrous-network nanostructures, hydrophilicity, high porosity, high water-holding capacity, high mechanical strength, crystallinity, and biocompatibility (Figure 2a) [49,50,51,52].

BC can be obtained by microbial fermentation (Figure 2b) and the cell-free culture system (Figure 2c) [53]. For microbial fermentation, the most famous BC-producing bacterium is *K. xylinus*, formerly known as *A. xylinum*. It is a Gram-negative, highly aerobic bacterium known as acetic acid bacteria (AAB). These bacteria grow at 25–30 °C with pH ranges from 3 to 7, using glucose as the best energy source [43]. In addition, BC can also be produced by *Bacillus*, *Leifsonia*, *Salmonella*, *Erwinia*, *Enterobacter*, *Pseudomonas*, and *Shewanella* [39]. Moreover, many genetically engineered bacteria have been constructed for enhancing bacterial cellulose production, improving the properties of BC and adding new functionalities to BC [54]. For example, Sajadi et al. expressed the cellulose synthase subunit D (*bcsD*) gene of *G. xylinum* BPR2001 in the wild-type *E. coli* Nissle 1917 to obtain bacterial cellulose with an increased crystallinity index [55]. In their another work, *bcsA* and *bcsB* genes from *G. xylinum* were expressed in *E. coli* Nissle 1917 to improve the yield of BC without influencing the crystallization index [56]. Although BC possesses various excellent properties as a useful natural hydrogel material, large-scale production of BC by microbial fermentation is challenging due to the high cost of the culture media, long fermentation time, and a large-scale fermentation facility [57]. Therefore, the development of novel bacterial cellulose production platforms that are not subject to the above limitations is important to facilitate the large-scale production and application of bacterial cellulose. The cell-free system is developing as an important in vitro platform for synthetic biology to produce various valuable substances using cell extracts (rather than microbial cells) [58], since the biosynthesis, regulation, and secretion process of substances are not impaired by cell walls or membranes. The cell-free system provides an efficient and economic platform for economically producing BC. For example, the cell-free system from *Gluconacetobacter hansenii* PJK that contains ATP and NADH utilizes glucose to synthesize cellulose with higher yield and glucose conversion efficiency than bacterial cells [59]. Moreover, it has been reported that cell-free cultures can reduce the cost of the synthetic process, decrease metabolic inhibitors, and maximize the rate of enzymatic reactions, thereby improving the efficiency and specificity of the biochemical reaction and expanding the application of the biochemical process.

### 2.2. Bacterial Cellulose Nanofibers

BCNFs are obtained through special treatments of bacterial cellulose, such as superfine, soft homeogenesis, and electrostatic spinning [60]. The diameter of a BCNF is smaller than that of bacterial cellulose, with a width range of 20 nm to 100 nm [61]. BCNFs have high surface area, high immobilization efficiency, minimal sensory impact when applied in food products, and protective effects when used as carrier materials [62]. Several strategies have be used to prepare BCNFs, such as homogenization [63], electrospinning [64], wet-drawing and wet-twisting [65], etc. [66].

### 2.3. Carboxymethyl Cellulose (CMC)

CMC is probably the best-known anionic linear polysaccharide derived from cellulose by reacting the sodium monochloroacetate with cellulose in an alkaline medium [67]. CMC is highly viscous and is generally considered non-toxic, non-allergenic, and biodegradable [68]. The degree of substitution (DS) of CMC affects the solubility of CMC molecules and the nature of the CMC solution [69]. CMC with DS values of 0.0–0.4 is insoluble in water but can undergo swelling. In contrast, CMC with DS values of 0.4–3 is soluble in water.

Cellulose (the precursors of CMC) can be obtained from a range of renewable biomass, including wood, cotton, crops, agricultural wastes, bacteria, and algae [70]. Currently, great efforts have been made to prepare CMC using green and eco-friendly methods to reduce environmental pollution and meet sustainable development requirements. For example, Moussa et al. prepared a series of CMC samples with different degrees of substitution by one-step carboxymethylation reaction of the celluloses extracted from almond stems, almond shells, and fig stems [71]. Besides cellulose, lignocellulosic materials can also be used to fabricate CMC. Akhlaq et al. used lignocellulosic wastes (eg., rice straw (RS), wheat straw (WS), sugarcane bagasse (SCB), and banana leaves (BL)) as raw materials to extract cellulose for CMC preparation [72]. Various CMCs from different raw materials are found to possess different physicochemical or morphological characteristics (e.g., DS, rheological properties, viscosity, water retention, and oil retention). For example, CMC obtained from wheat straw possessed a DS value of 2.1, and the DS value of CMC from banana leaf waste was 0.7 [72]. Moreover, CMC can also be obtained from cellulose-containing wastes such as those from the textile industry (e.g., knitted rags or cotton lint) and household/office products (e.g., office wastepaper, paper sludge, or waste textiles). Employing these waste materials to produce CMC will lower the production costs and mitigate environmental pollution [73].

### 2.4. Cellulose Nanofiber (CNF)

CNFs are crystalline fibers with a length of >1 μm and a width of 5–200 nm, consisting of bundles of fibrils, and are mainly isolated from wood or plant sources such as cotton, flax, bamboo, hemp, etc. [74]. Based on their excellent biocompatibility, renewability, biodegradability, and the properties (e.g., high crystallinity, high specific surface area, and good rheological behavior) derived from their nanostructure, CNFs have attracted enormous interest in many different fields over the past decades. CNF is also used as a cellulose-based material for probiotic encapsulation to improve the survival rate of probiotics during processing, storage, and delivery processes. Pretreatment is critical for efficient and sustainable CNF production. Several methods can be used to pretreat cellulose to produce CNF, such as enzyme treatment, mechanical pretreatment (e.g., high-pressure homogenization, ball milling, cryocrushing, microfluidization, and ultra-fine grinding), and chemical pretreatment [75]. Each pretreatment method has advantages and disadvantages when applied to the production of CNF [76]. For example, enzyme pretreatment is effective in removing hemicellulose and lignin from lignocellulosic feedstocks, resulting in smaller CNF diameter and increased crystallinity and homogeneity after fibrillation. However, enzymatic pretreatment must be carefully controlled to avoid excessive enzymatic hydrolysis, as it breaks down cellulose components and reduces overall fiber length and DP. In addition, the high cost of enzymes limits their use in industry. Mechanical pretreatment requires high energy consumption. Chemical pretreatment requires several washing steps due to the use of various chemical solvents, and may bring some environmental problems [77].

In general, the low cost, easy availability, and biocompatibility of cellulose make it useful in various fields. Moreover, cellulose can be modified to endow it with multiple properties and functions, and thus enhance the protective effect of cellulose on the encapsulated probiotics (Table 2).

## 3. Probiotic Encapsulation Strategies with Cellulose-Based Materials

### 3.1. Electrospinning

Electrospinning is an electro-liquid droplet kinetic process in which micro-jets of liquid droplets formed under the influence of an electric charge are mechanically stretched, elongated, and cured by drying steps into fibers and adsorbed onto a receiver plate, creating continuous polymer fibers with diameters ranging from nanometer to micron scale [83]. The electrospinning device consists of a syringe push injection pump, a spinneret, a high voltage power supply, and a collector. During the electrospinning process, the polymer solution is pumped into the spinneret, causing the droplets to be charged and resulting in a surface repulsion greater than the surface tension. Then, the droplets take on a conical structure, producing a Taylor cone [84], where the jet is initially extended in a straight line, followed by a violent whipping motion due to bending instability, and rapidly solidifies when the jet is stretched to a finer diameter, thus resulting in the deposition of solid fibers onto the grounded collector [85].

**Figure 3:**
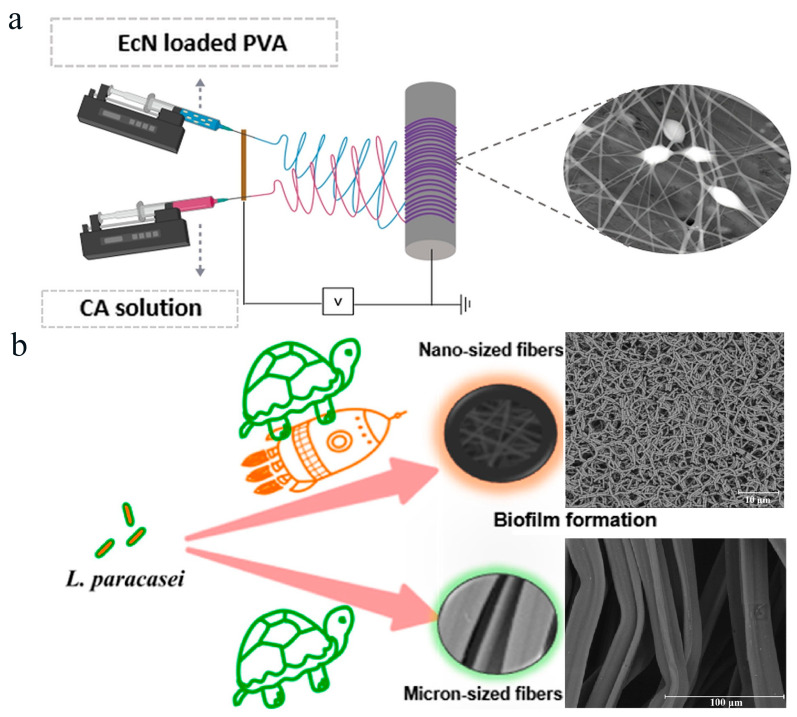
Probiotics encapsulated in electrospun cellulose-based film. (**a**) Novel cellulose acetate (CA) and polyvinyl alcohol (PVA) mixed fibers prepared by inclined double-nozzle electrospinning. Adapted from Ref. [36], with permission of Elsevier Ltd., 2021. (**b**) Electrospun acetate nanofiber membrane for the enrichment of *L. paracasei* biofilms. Adapted from Ref. [87], with permission of American Chemical Society, 2021.

Fibers can be fabricated by electrospinning to encapsulate probiotics for protecting them from adverse environments (e.g., temperature, oxygen, water activity, pressure, pH, hydrogen peroxide, digestive enzymes, etc.) and ensuring no interaction with other components of the matrix [84]. The physicochemical properties of the polymer solution used for forming the film are critical to the formation of the fibers and the effectiveness of probiotic encapsulation. Çanga et al. prepared novel cellulose acetate (CA) and polyvinyl alcohol (PVA) hybrid fibers for encapsulating probiotics using a two-nozzle electrospinning method to improve the gastrointestinal stability of probiotics (Figure 3a) [36]. In that work, *E. coli* Nissle 1917 (EcN) was encapsulated in the PVA/CA composite film, where CA enhanced the stability of the probiotic under gastric conditions and PVA provided a protection effect against toxic solvents during electrospinning. As can be seen from the ESEM images, EcN cells were successfully encapsulated in PVA/PVA fibers and PVA/CA fibers during the electrospinning process. The survival rate of probiotics under simulated gastrointestinal conditions was significantly improved after encapsulation with PVA/CA fibers. The above research cleverly utilized the unique characteristics of CA and used it for the first time to encapsulate probiotics in electrospun mixed fibers. This study provided an angled dual-nozzle electrospinning method for improving the survival rate of probiotics in the gastrointestinal tract using PVA/CA fibers. In addition to encapsulating probiotics by mixing them with a cellulose-based solution as an electrospun liquid, electrospun cellulose films can also be used as a culture support for culturing probiotics into biofilms. It has been reported that biofilms possess excellent resistance to harsh environments, and probiotic biofilms can endow probiotics with excellent resistance and adhesion abilities in the intestine [86]. Meng et al. studied the electrospun cellulose acetate nanofibrous membrane as a probiotic biofilm enrichment material. They found that electrospun cellulose acetate nanofibrous membranes exhibited outstanding advantages in the enrichment of *Lactobacillus paracasei* (*L. paracasei*) biofilms compared with micrometer scale fiber membranes. Overall, electrospun cellulose acetate nanofibrous membrane is an ideal material for enriching probiotic biofilms and has certain development potential (Figure 3b) [87].

### 3.2. Cross-Linking

**Figure 4:**
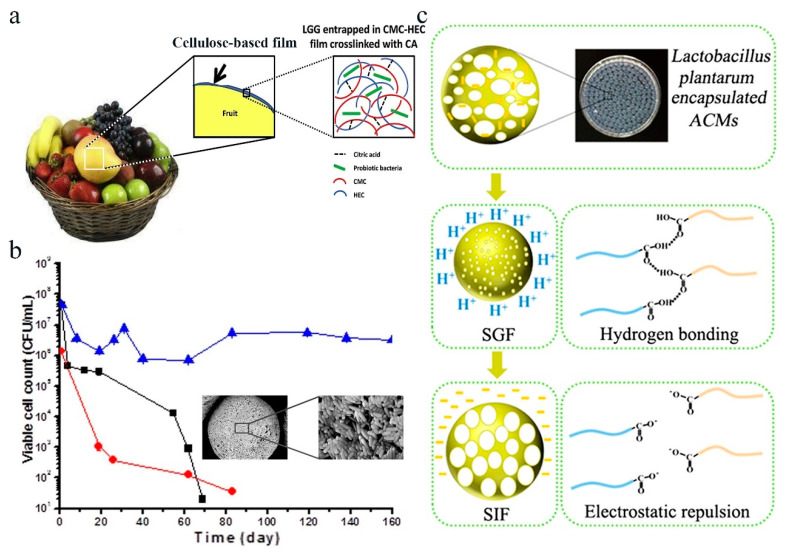
Probiotic encapsulation by cross-linking method. (**a**) Preparation of cellulose-based encapsulation films by cross-linking CMC and HEC with CA. Adapted from Ref. [88], with permission of Elsevier Ltd., 2019. (**b**) The enhanced storage stability of probiotics after ACFP composite capsule encapsulation. Black: AG, Ca-alginate gel with *L. plantarum*. Red: ACG, Ca-alginate/cellulose gel with *L. plantarum*. Blue: ACFP, Ca-alginate/cellulose/cryoprotectant gel with *L. plantarum*. Adapted from Ref. [37], with permission of Elsevier Ltd., 2019. (**c**) Scheme illustrating the morphology of ACMS encapsulated probiotics and the pH-responsive mechanisms. Adapted from Ref. [80], with permission of American Chemical Society, 2018.

Another strategy for fabricating probiotic cellulose films is cross-linking. During the cross-linking process, the cellulose macromolecules interact with bifunctional molecules, resulting in the formation of cross-linked bonds (e.g., bridge bonds) between cellulose macromolecules in a web-like structure. The selection of different types of cellulose and suitable cross-linking agents to prepare cellulose films can seal probiotics in them and improve the stability of probiotics, showing promising applications in different fields. Sodium carboxymethyl cellulose (CMC) and hydroxyethyl cellulose (HEC) were used to fabricate cellulose-based films with citric acid (CA) as the cross-linking agent (Figure 4a) [88]. It was found that the thickness of the film depended on the amount of CA. As the concentration of CA increased, the rigidity increased, while the swelling rate and the surface area of the film decreased. However, high CA content induced the formation of fibrous aggregates in the membranes and led to structural defects. The prepared cellulose membranes can effectively encapsulate live *Lactobacillus rhamnosus* GG (*L. rhamnosus*) (LGG) in the membrane after soaking in bacterial media. With the protective effect of the cellulose membrane, the survival rate of LGG was greatly improved. In another study, Li et al. prepared Ca-alginate/cryoprotectants/cellulose composite (ACFP) capsules to encapsulate *L. plantarum* (Figure 4b) [37]. After encapsulation, the storage stability, and the survival rate of *L. plantarum* during a vacuum freeze-drying process were greatly improved. Furthermore, in addition to the protective effect of the encapsulation matrix, controlled release of probiotics is important for probiotics to have efficient effects. In this respect, sodium alginate (SA) is a good choice due to the pH-responsive property of SA. Zhang et al. used sodium alginate (SA) and TEMPO oxidized cellulose nanofiber (CNF) to prepare alginate/cellulose nanofiber gel microspheres (ACM) loaded with *L. plantarum* by a cross-linking reaction of calcium ions (Figure 4c) [80]. It was found that the acidic environment of the human stomach would stabilize the gel macrospheres by reducing electrostatic repulsion and forming hydrogen bonds between SA and CNF, protecting *L. plantarum* cells to a greater extent. In contrast, the neutral simulated intestinal fluid dissolved the gel macrospheres and facilitated the release of *L. plantarum* cells in the human intestine. This work provides a pH-responsive encapsulation method for realizing the controlled release of probiotics, showing potential application in the intestine-targeted delivery of probiotics.

### 3.3. In-Situ Growth

**Figure 5:**
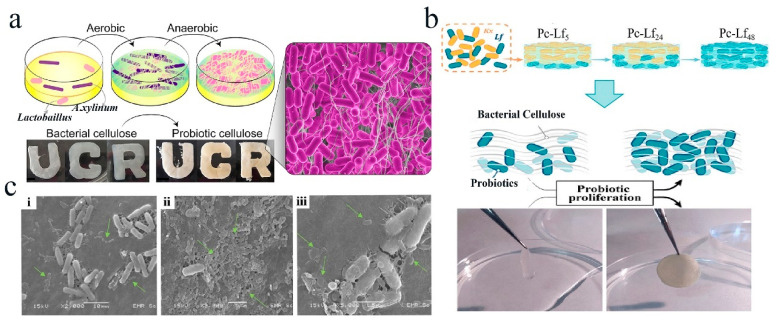
Probiotics films prepared by in-situ growth method. (**a**) Graphic illustrating the preparation process of probiotic cellulose by co-culturing *Ax* and probiotics. Adapted from Ref. [89], with permission of Elsevier BV, 2021. (**b**) Scheme illustrating the preparation of PC-*Lf* by co-incubating *Kx* and *Lf*. Adapted from Ref. [90], with permission of American Chemical Society, 2022. (**c**) Electron micrographs of the freeze-dried KBC incorporated with *L. plantarum* TISTR 541 cells by the adsorption–incubation method. (i–iii) *L. plantarum* attached on KBC fibrils at different magnifications (Green arrows represent *L. plantarum*). Adapted from Ref. [38], with permission of MDPI, 2023.

In addition to the above-mentioned encapsulation strategies, probiotics can also be incorporated into the BC network structure by co-culturing *A. xylinum* (*Ax*) and probiotics to form probiotic cellulose [89]. This encapsulation method not only increases the survival rate of the probiotics but also endows BC with multifunctional properties. Moreover, this method is simple and environmentally friendly without using any toxic agents. For example, Sabio et al. obtained a probiotic cellulose for treating severe skin infections and chronic wounds by co-culturing *Ax* suspensions and probiotics (*Lactobacillus fermentum* (*Lf*) or *Lactobacillus gasseri* (*Lg*)) (Figure 5a) [89]. In their work, *Ax* and *Lf* or *Lg* suspensions were co-cultured in a 1 mL Hestrin–Schramm (HS) medium at a volume ratio of 1:1 and co-cultured at 30 °C under aerobic conditions. Bacterial cellulose containing *Ax* and *Lf* or *Lg* was obtained after 3 days’ incubation. Afterwards, the HS medium was replaced by a MRS (5 mL) medium, and the bacterial suspensions were anaerobically incubated at 37 °C for 48 h. After incubation for 24 h, the MRS medium was replaced with fresh MRS, and probiotic-cellulose films (*Lf*- or *Lg*-cellulose) were obtained after 48 h incubation in MRS. This encapsulating strategy of co-culturing BC producing strains with probiotics to obtain probiotic cellulose by regulating the oxygen conditions without expensive and toxic chemical treatments provides a good idea for developing BC-embedded probiotics. In their another work, the authors prepared a cellulose-based material by embedding probiotics to modulate the cellulose viscoelasticity via the probiotic proliferation. It was found that this living cellulose-based material possessed lower-than-matrix viscoelasticity at a low probiotic density, and became an elastic solid promoted by the probiotic proliferation. Therefore, it can be used as a biological ink and applied in the field of 3D printing (Figure 5b) [90]. In addition to BC produced from *Ax*, Charoenrak et al. produced Kombucha bacterial cellulose (KBC) by fermentation of Kombucha, which is mainly composed of tea and sugar as the nutrients, to protect *L. plantarum* (Figure 5c) [38]. In summary, the in-situ growth method for producing bacterial cellulose is simple, easy to operate, eco-friendly, and does not require the use of toxic chemical reagents. Additionally, probiotics can be incorporated into bacterial cellulose through co-cultivation, providing an effective and convenient strategy for encapsulating probiotics.

### 3.4. Casting

**Figure 6:**
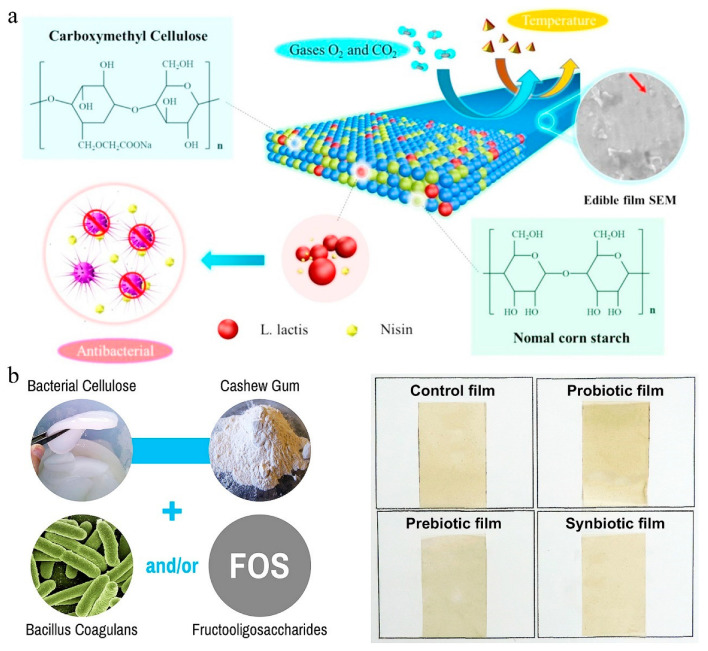
Probiotic films prepared by casting. (**a**) Preparation of carboxymethyl cellulose based probiotic film by casting (the red arrow represents the probiotic bacteria). Adapted from Ref. [31], with permission of Elsevier Ltd., 2020. (**b**) The spore-producing drug-resistant bacteria (*B. coagulans)* combined with a biopolymer mixture (bacterial cellulose—BC and cashew gum—CG) as a carrier substrate, four different films were produced by casting. Adapted from Ref. [91], with permission of Elsevier Ltd., 2020.

Casting is also a common method for encapsulating probiotics into cellulose membranes [64]. Lan et al. used a casting method to produce a composite film composed of corn starch (NS) and carboxymethyl cellulose (CMC) embedded with *Lactobacillus lactis* (*L. lactis*) (Figure 6a) [31]. In this work, CMC powder was added to 1.5% NS solution to fix the total concentration of NS and CMC at 3% (NS and CMC were added in a ratio of 5:5), and 1.5% (wt%) glycerol was added to the solution. Then, *L. lactis* with a final concentration of 1.5% was mixed with the above solution. Finally, the above mixtures were poured onto a glass plate and dried at 28 °C to obtain the composite film. The prepared film can inhibit the growth of *Staphylococcus aureus* due to the release of nisin by *L. lactis*. These antibacterial films promise to be used in low-moisture food packaging in the future. Alcântara et al. produced different carboxymethyl cellulose-based films containing *Bacillus coagulans* (*B. coagulans*) by a casting method to improve the storage stability of probiotics (Figure 6b) [91]. In this study, FOS was incorporated into the film to improve the viability of *B. coagulans*. In another study, El-Sayed et al. used chitosan (CH), SA, CMC, and microcrystalline cellulose (AMCC) to prepare a fiber to encapsulate probiotic strains (*Bifidobacterium lactis*, *Lactobacillus acidophilus* (*L. acidophilus*) and *Lactobacillus casei* (*L. casei*)) by a casting method [32]. Such films protected probiotics from gastrointestinal digestion and maintained a sufficient quantity and activity of the probiotics. Mozaffarzogh et al. prepared a carboxymethyl cellulose (CMC)-sodium caseinate (SC)-based film by a homogenizing method to encapsulate *L. acidophilus*, *Lactobacillus reuteri*, *L. casei*, *L. rhamnosus,* and *Bifidobacterium bifidum* [92]. The obtained probiotic film can be used for food packaging to extend the shelf life of sturgeon fillets.

In summary, four encapsulation strategies, namely, electrospinning, cross-linking, in-situ growth, and casting, are extensively discussed in this section (Table 3). The four packaging methods have their inherent advantages and disadvantages. The preparation efficiency of the electrospinning method is high, and a variety of fiber structures and shapes can be prepared. However, this method also has certain limitations, requiring complex electrospinning equipment, and the fiber prepared by this method is uneven in thickness. The cross-linking method can improve the mechanical properties of the fiber and enhance the stability of the fiber, but excessive cross-linking will cause fiber aggregation. In-situ growth is easy to operate and does not require the use of toxic chemicals, but it takes a long time to produce fibers and is difficult to remove the microorganisms used to produce cellulose fibers. The casting method can improve the physical properties of the prepared materials, but the operation process is complicated and cumbersome. Currently, some patented encapsulated probiotics have been reported based on electrospinning, cross-linking, and spray drying (Table 4). However, compared with the other three encapsulation methods, the in-situ growth encapsulation method is currently less researched and is hardly used for the production of patent encapsulated probiotics.

## 4. Cellulose-Based Probiotic Films for Food Applications

### 4.1. Food Packaging

**Figure 7:**
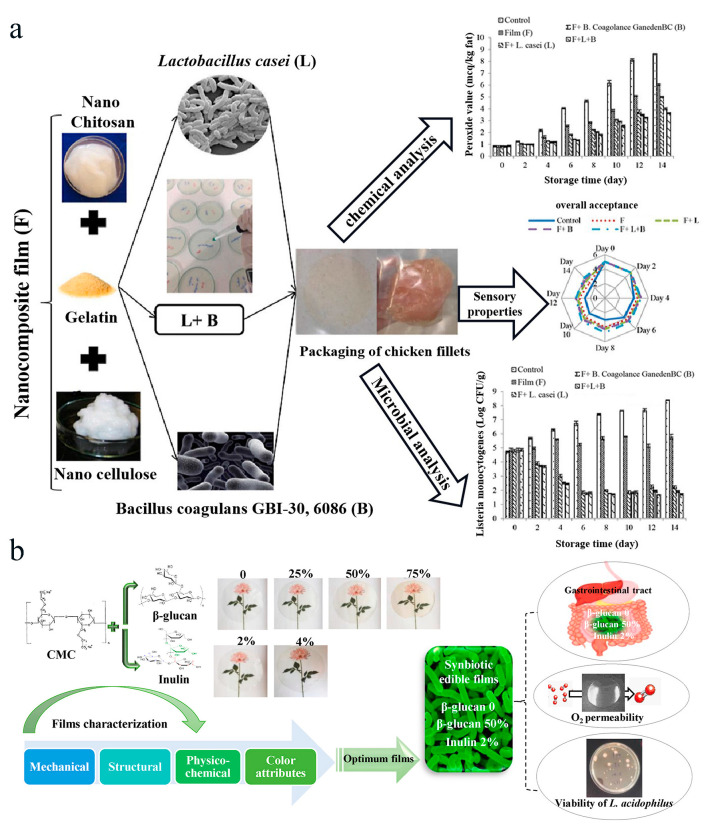
Application of cellulose encapsulated probiotics in food packaging. (**a**) Nanocellulocellulose-nano-chitosan gelatin film was used to encapsulate *L. casei* and *B. coagulans* GBI-306086 and their combination for preservation of meat products. Adapted from Ref. [35], with permission of Elsevier Ltd., 2022. (**b**) Based on CMC/βMglucan (BG), *L. acidophilus* LA-5 was prepared according to four ratios of 100:0, 75:25, 50:50, and 25:75, and different proportions of inulin (IL, 2%, 4%) were added to predict the shelf life of food. Adapted from Ref. [34], with permission of Elsevier BV, 2022.

Food packaging plays an important role in protecting food from chemical, physical, or microbial contaminations to extend the shelf life of food and ensure food safety [27]. Conventional packaging materials come from petroleum-based plastic, which have brought serious environmental pollution problems due to their non-biodegradability and the release of toxic substances during the recycling process. Cellulose-based materials have attracted great attention in food packaging due to their biodegradable, environmentally friendly, and easily accessible properties. Entrapment of probiotic cells in cellulose-based films can make the packaging bioactive due to the high antimicrobial ability of the probiotics and their metabolites [29,31,33,100,101]. For example, Moghanjougi et al. fabricated a bacterial cellulose film containing free or microencapsulated probiotics (*L. acidophilus* or *Bifidobacterium animalis*) to inhibit the growth of *Aspergillus niger* in cheese [29]. The results showed that bacterial cellulose films containing probiotics microencapsulated with SA maintained a high viability of the probiotics and were most effective in inhibiting the growth of *Aspergillus niger* in cheese. Moreover, prebiotics can be incorporated into cellulose-based probiotic films to further increase the survival rate of probiotics. For example, Zabihollahi et al. prepared a CMC-based cellulose film to encapsulate probiotic bacteria (*L. plantarum*) for extending the shelf life of chicken tenders. In this system, inulin was added to the film, enhancing the survival rate of probiotics by 36% [33]. Salimiraad et al. constructed novel probiotic cellulose-based films for preserving fresh chicken fillets (Figure 7a) [35]. In this system, the cellulose-based films were prepared by mixing nano cellulose, nano chitosan, gelatin, and probiotics (e.g., *L. casei*, *B. coagulans*, and their combinations). The results showed that the nanocomposite probiotic film could extend the shelf life of the frozen fish fillets by inhibiting the growth of *listeria monocytogenes*. In addition to probiotics, synbiotics, which contain probiotics and prebiotics are of great interest due to their excellent health-promoting capacities [102]. Food packaging materials incorporated with synbiotics are of great interest to researchers. Seyedzadeh-Hashemi et al. prepared cellulose-based films containing synbiotics composed of β-glucan, inulin, and *L. acidophilus* [34]. In this system, the addition of inulin effectively improved the extensibility and the oxygen barrier property of the films. In addition, the incorporation of *L. acidophilus* LA-5 with inulin into the CMC/BG film resulted in a much higher stability under storage and simulated gastrointestinal conditions. Collectively, the cellulose-based probiotic films can not only protect probiotics from harsh processing environments and prolong probiotic stability but also provide a new platform for bioactive packaged foods (Figure 7b). Although considerable fundamental research related to cellulose-based probiotic encapsulation has been reported, moving the practical food applications of probiotic-containing cellulose films from lab to food applications seems to be a difficult task at present, possibly due to the fact that the cost of the film may exceed the cost of the packaged food due to the high cost of the preparation technologies of the cellulose-based probiotic films. For example, electrospinning equipment is complex, large-scale production of electrospun probiotic film requires a large amount of expensive equipments and the equipment needs to be maintained in the later stage, resulting in very high production costs, which are not conducive to the industrialization of products. If we want to apply cellulose-based probiotic film to other food packaging, it is possible to choose to improve the main structure of the cellulose-based probiotic film, to optimize the size and thickness of the film, to simplify the preparation process, to shorten the preparation time, and to reduce the consumption of energy and materials. In summary, there is still a need to continuously explore appropriate encapsulation strategies with cellulose or cellulose-based materials, to develop suitable cellulose preparation technologies, and to develop new types of probiotics to facilitate the practical application of cellulosed-based probiotic films.

### 4.2. Food Manufacturing

**Figure 8:**
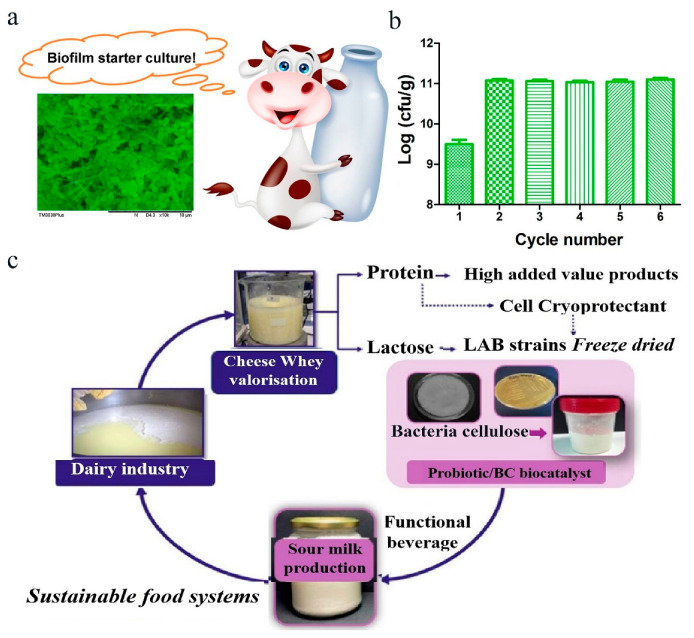
Application of cellulose encapsulated probiotics in food manufacturing. (**a**) Probiotic biofilm fermentation of milk. Adapted from Ref. [21], with permission of American Chemical Society, 2019. (**b**) Viable cells in the fermented milk with the recycled lyophilized powders of *L. plantarum* biofilms in six batches. Adapted from Ref. [21], with permission of American Chemical Society, 2019. (**c**) The lactic acid bacteria (LAB) culture was fixed on bacterial cellulose (BC) as a biological starter. Adapted from Ref. [30], with permission of MDPI, 2022.

In addition to food packaging, cellulose-based films can be used as scaffolds for the formation of microbial biofilms or as starter cultures and bioreactors to produce fermented products and valuable metabolites. For example, Hu et al. prepared electrospun nanofiber-based films for probiotic biofilm growth. The electrospun nanofibers embedded with probiotic biofilms can be used as the starter culture for the fermentation of milk (Figure 8a) [21]. The electrospun nanofiber membranes were proven to be an excellent scaffold for *L. plantarum* biofilm growth. Probiotic biofilms on the electrospun nanofiber membranes showed excellent gastrointestinal resistance compared with the planktonic bacteria. When the probiotic biofilm-containing membranes were used as the starter culture to produce fermented milk, they showed excellent fermentative ability with a decreased fermentation time and a higher survival rate of *L. plantarum* during the shelf life. Moreover, the number of surviving bacteria in the fermented milk after the first batch increased to approximately 11.06 log CFU/g in the recycled batches and remained at this level throughout the tested reusable batches (Figure 8b). In another study, Lappa et al. prepared a bacteria cellulose (BC) film as a novel biocatalyst for producing functional sour milk (Figure 8c) [30]. In this work, *Lactiplantibacillus pentosus* B329 and *Lactiplantibacillus plantarum* 820 were selected as starter cultures. After the encapsulating of bacterial strains with BC, the cell viability and metabolic activity were sustained. Moreover, the probiotic viability during storage was improved after immobilization on BC, and the fermented milk produced by the BC-encapsulated lactic acid bacteria (LAB) displayed better organoleptic properties. This work presents cellulose-based platforms for encapsulating LAB used in fermentation for improving the survival rate of LABs during processing and extending the stability of the fermented products.

Collectively, the application of cellulose-based probiotic film in the food field is useful and involves many aspects, including food packaging and biocatalysts. Since probiotics are added to cellulose-based films to endow them with antibacterial ability and to prolong shelf life, most cellulose-based probiotics films are used as food packaging films. However, probiotic cellulose membrane containing a shelf-life prediction function is less studied and should be further developed. In addition, cellulose-based probiotic films have the potential to be used as biocatalysts, and more cellulose-based probiotic membranes should be extended in this direction in the future.

## 5. Conclusions and Perspective

In this review, we summarized the types of cellulose materials, including bacterial cellulose, bacterial cellulose nanofibers, carboxymethyl cellulose, and cellulose nanofibers used for encapsulating probiotics. The preparation methods for cellulose-based films were also briefly reviewed in relation to electrospinning, cross linking, in-situ growth, and casting strategies. Furthermore, we summarized the probiotic-encapsulating strategies using cellulose-based films. Finally, the applications of cellulose-based-film encapsulated probiotics were also discussed, with emphasis on their applications in food packaging and food manufacturing. In addition, to summarize the previous studies of cellulose-based probiotic films, we also propose some unsolved problems of the current studies about cellulose-based film for encapsulating probiotics and put forward several future research directions in this field.

Although many efforts have been devoted to developing cellulose-based films for probiotic encapsulation and applying the encapsulated probiotics in various food fields in the past years, some unsolved issues still exist and need to be addressed:

(1) The properties and functions of probiotics after different encapsulations should be further explored, and the action mechanisms of the encapsulated probiotics should also be continuously explored and accurately elucidated. Moreover, advanced strategies for fabricating cellulose-based materials for the encapsulation of probiotics should be developed, such as 3D or 4D printing.

(2) The safety, environmental friendliness, reusability, and biodegradability of cellulose-based probiotic films should be evaluated. Moreover, the relationship between the probiotics and the packaged food should be further evaluated.

(3) Most of the current research on developing cellulose-based probiotic films and their applications are still in the laboratory stage; large-scale production of cellulose-based probiotic films will further promote the practical application of the films. It is necessary to develop appropriate encapsulation strategies that are suitable for large-scale production of cellulose-based probiotic films. Moreover, the selection of easily processable and cost-effective cellulose and probiotics also influences the cost of the probiotic films.

(4) The shelf-life prediction cellulose film containing probiotics should be widely developed and applied.

Despite the above-mentioned challenges, it is expected that cellulose-based probiotic films will play a vital role in future food preservation. It is also hoped that this review will inspire future researchers to develop more effective and economic strategies for the production of cellulose-based probiotic films and promote their successful large-scale fabrication and practical applications.

## Figures and Tables

**Table 1:** Types of cellulose used for probiotic encapsulation and their applications.

Cellulose Type	Probiotic Type	Survival Rate of Probiotics before Cellulose Encapsulation	Survival Rate of Probiotics after Cellulose Encapsulation	Application of the Cellulose-Based Probiotic Films	Ref.
Bacterial cellulose	*Lactobacillus acidophilus*, *Bifidobacterium animalis*	–	–	Bio-preservation	[29]
Bacterial cellulose	*Lactiplantibacillus pentosus*, *Lactiplantibacillus plantarum*	Less than 80% (After 5 months of storage at 4 °C)	About 90–95% (After 5 months of storage at 4 °C)	Milk fermentation	[30]
Bacterial cellulose nanofibers	*Lactobacillus plantarum*	<60% (Treatment in pH 2.5, 3.5, 4.5 and 6.8 for 3 h)	>150% (Treatment in pH 2.5, 3.5, 4.5 and 6.8 for 3 h)	Milk fermentation	[21]
Carboxymethyl cellulose	*Lactobacillus lactis*	–	–	Improving nisin production	[31]
Carboxymethyl cellulose	*Bifidobacterium lactis*, *Lactobacillus acidophilus*, *Lactobacillus casei*	The number of probiotics is less than 7.00 log CFU/g (45 days of storage at 7 °C)	The number of probiotics exceeded 8.00 log CFU/g (45 days of storage at 7 °C)	Food coating	[32]
Carboxymethyl cellulose	*Lactobacillus plantarum*	–	–	Bioactive food packaging	[33]
Carboxymethyl cellulose	*Lactobacillus acidophilus*	About 49% (Digest in simulated gastric juices for 120 min)	About 70% (Digest in simulated gastric juices for 120 min)	Antibacterial food coating	[34]
Cellulose nanofiber	*Lactobacillus casei*, *Bacillus coagulans*	–	–	Food packaging	[35]
Cellulose acetate	*Escherichia coli* Nissle 1917	0% (Digest in a simulated digestive system for 100 min)	About 26% (Digest in a simulated digestive system for 100 min)	–	[36]
Cellulose microgels	*Lactobacillus plantarum*	The number of viable bacteria decreased by 10^5^ (Freeze drying)	The number of viable bacteria decreased by 10^3^ (Freeze drying)	–	[37]
Kombucha bacterial cellulose	*Lactobacillus plantarum*	About 33% (Freeze drying)	About 49% (Freeze drying)	Antibacterial food packaging	[38]

**Table 2:** Grafted cellulose for the encapsulation of probiotics.

Probiotic Type	Cellulose Type	Grafting Method	Function	Ref.
*Lactobacillus plantarum*	Cellulose nanofiber	TEMPO-mediated oxidation to endow cellulose with carboxyl groups	Improving the survival rate and the intestinal retention time of the probiotics	[78]
*Lactobacillus casei*	Cellulose	Sulfation of cellulose to endow it with negatively charged sulfuric acid groups	Improving the survival rate and intestinal delivery rate of the probiotics, realizing their controllable release	[79]
*Lactobacillus plantarum*	Cellulose nanofiber	TEMPO-mediated oxidation	Improving the survival rate of the probiotics and realizing the controllable release of the probiotics	[80]
*Bifidobacterium adolescentis* and *Bacillus subtilis*	Carboxymethyl cellulose	Obtaining mercaptoylcarboxymethyl cellulose through the EDC (1-ethyl-3(3-dimethylaminopropyl-carbodiimide hydrochloride)/NHS (N-hydroxysuccinimide) chemistry	Improving the survival rate and improving the storage stability of the probiotics and promoting the proliferation, adhesion, and colonization of probiotics	[81]
*Saccharomyces cerevisiae*	Cellulose nanocrystals	The complexation of shellac and cellulose nanocrystals via hydrogen bonding	Improving the survival rate of the probiotics and realizing the controllable release of the probiotics	[82]

**Table 3:** Encapsulation strategies for probiotics with cellulose-based materials.

Encapsulation Strategies	Cellulose Type	Probiotic Type	Advantage	Disadvantage	Refs.
Electrospinning	Cellulose acetate; Cellulose acetate nanofiber	*Escherichia coli* Nissle 1917; *Lactobacillus paracasei*	High preparation efficiency, a variety of fiber structures and shapes can be prepared	Uneven fiber thickness, and complex electrospinning equipment	[36,83,87]
Cross-linking	Carboxymethyl cellulose and hydroxyethyl cellulose; Cellulose; TEMPO oxidized cellulose nanofiber	*Lactobacillus rhamnosus*; *Lactobacillus plantarum*; *Lactobacillus plantarum*	Improving mechanical properties and enhancing the stability of the fiber	Fiber aggregation caused by excessive cross-linking	[37,80,88]
In-situ growth	Bacterial cellulose; Bacterial cellulose; Kombucha bacterial cellulose	*Lactobacillus fermentum* and *Lactobacillus gasseri*; *Lactobacillus fermentum*; *Lactobacillus plantarum*	It is easy to operate and does not require the use of toxic chemicals	Long production period, and difficult to remove the microorganisms used for producing cellulose fiber	[38,89,90]
Casting	Carboxymethyl cellulose; Carboxymethyl cellulose; Carboxymethyl cellulose and microcrystalline cellulose; Carboxymethyl cellulose	*Lactobacillus lactis*; *Bacillus coagulans*; *Bifidobacterium lactis*, *Lactobacillus acidophilus* and *Lactobacillus casei*; *Lactobacillus acidophilus, Lactobacillus reuteri*, *Lactobacillus casei*, *Lactobacillus rhamnosus* and *Bifidobacterium bifidum*	It can improve the physical properties of materials	Complicated and tedious operation process	[31,32,91,92]

**Table 4:** Patented encapsulated probiotics.

Encapsulation Materials	Encapsulation Method	Suggested Application	Patent Number	Ref.
Sodium alginates/PEG 4000/methacrylate polymers	Ion gelation	Drugs that promote intestinal health	IN201711011030A	[93]
Casein/starch	Spray drying	Probiotic powder supplement	US8871266B2	[94]
4-(2-hydroxyethyl)-1-piperazine-ethanesulfonic acid/glycine/betaine/carboxymethyl cellulose	Cross-linking	A drug to treat intestinal disorders	WO2018230939A1	[95]
Sodium carboxymethyl cellulose/maltodextrin	Freeze drying	Freeze-dried powder preparation of probiotics	US11571387 B2	[96]
Millet extract powder	Freeze drying or spray drying	Functional food supplements or dietary supplements	US10576113B2	[97]
Gum Arabic/polyvinyl alcohol/polyvinylpyrrolidone/whey protein concentrate or maltodextrin	Electrospinning	Probiotic capsules	US2023/019354 A1	[98]
Monovalent alginate/gelatin or cellulose	Freeze drying or spray drying	Probiotic powder supplements	US2004/0175389 A1	[99]
